# The use and future perspective of Artificial Intelligence—A survey among German surgeons

**DOI:** 10.3389/fpubh.2022.982335

**Published:** 2022-10-05

**Authors:** Mathieu Pecqueux, Carina Riediger, Marius Distler, Florian Oehme, Ulrich Bork, Fiona R. Kolbinger, Oliver Schöffski, Peter van Wijngaarden, Jürgen Weitz, Johannes Schweipert, Christoph Kahlert

**Affiliations:** ^1^Department of Visceral, Thoracic and Vascular Surgery, University Hospital Carl Gustav Carus Dresden, Technische Universität Dresden, National Center for Tumor Diseases Dresden (NCT/UCC), Dresden, Germany; ^2^Else Kröner Fresenius Center for Digital Health (EKFZ) Dresden, Faculty of Medicine Carl Gustav Carus, Technische Universität Dresden, National Center for Tumor Diseases Dresden (NCT/UCC), Dresden, Germany; ^3^Chair of Health Management, Friedrich-Alexander-Universität Erlangen-Nürnberg, Nürnberg, Germany; ^4^Centre for Eye Research Australia, Royal Victorian Eye and Ear Hospital, Melbourne, VIC, Australia; ^5^Ophthalmology, Department of Surgery, University of Melbourne, Melbourne, VIC, Australia; ^6^German Cancer Consortium (DKTK), Partner Site Dresden, German Cancer Research Center (DKFZ), National Center for Tumor Diseases Dresden (NCT/UCC), Heidelberg, Germany

**Keywords:** artificial intelligence, acceptance, surgery, survey, perspective

## Abstract

**Purpose:**

Clinical abundance of artificial intelligence has increased significantly in the last decade. This survey aims to provide an overview of the current state of knowledge and acceptance of AI applications among surgeons in Germany.

**Methods:**

A total of 357 surgeons from German university hospitals, academic teaching hospitals and private practices were contacted by e-mail and asked to participate in the anonymous survey.

**Results:**

A total of 147 physicians completed the survey. The majority of respondents (*n* = 85, 52.8%) stated that they were familiar with AI applications in medicine. Personal knowledge was self-rated as average (*n* = 67, 41.6%) or rudimentary (*n* = 60, 37.3%) by the majority of participants. On the basis of various application scenarios, it became apparent that the respondents have different demands on AI applications in the area of “diagnosis confirmation” as compared to the area of “therapy decision.” For the latter category, the requirements in terms of the error level are significantly higher and more respondents view their application in medical practice rather critically. Accordingly, most of the participants hope that AI systems will primarily improve diagnosis confirmation, while they see their ethical and legal problems with regard to liability as the main obstacle to extensive clinical application.

**Conclusion:**

German surgeons are in principle positively disposed toward AI applications. However, many surgeons see a deficit in their own knowledge and in the implementation of AI applications in their own professional environment. Accordingly, medical education programs targeting both medical students and healthcare professionals should convey basic knowledge about the development and clinical implementation process of AI applications in different medical fields, including surgery.

## Introduction

Artificial intelligence (AI) is a technology that aims at an imitation of the human ability to learn and to derive logical conclusions from large amounts of training data.

AI can be divided into four broad categories: Speech understanding (i.e., methods for speech and text recognition and text generation, e.g., using natural language processing technologies), image recognition (e.g., based on computer vision technologies), machine learning (e.g., based on neural networks), and knowledge-based systems (*via* the use of cognitive modeling or semantic technologies, among others).

In 2016, Grace et al. conducted a survey of 1,634 experts on AI applications and machine learning (1). A total of 352 researchers responded to the survey and indicated that AI applications will increasingly outperform humans in some complex tasks in many areas in the coming decades. This includes translating languages (by 2024), writing school essays (by 2026), driving a truck (by 2027), working in retail (by 2031), writing a best-selling book (by 2049), and working as a surgeon (by 2053) (1). Accordingly, interest in AI applications in medicine and especially in surgery has also grown significantly.

Although the use of artificial intelligence is not new, the recent pandemic has not only increased awareness for the digitalization of medicine such as virtual consultations, but also revealed and accentuated long existing staff shortages, thus reigniting the discussion about personnel relief through the use of artificial intelligence.

There are numerous potential applications for AI in medical field, among the most advanced applications for artificial intelligence are diagnostic procedures in the fields of radiology, pathology and dermatology. In a recent study, McKinney et al. investigated whether artificial intelligence can be used to improve the evaluation of mammography images (2). Although this procedure is now highly standardized and diagnosis is often performed in 4- or 6-eye fashion, the error rate for false-positive or false-negative findings nevertheless remains high (2, 3). An AI system on a series of screening mammograms from more than 30,000 women from the United Kingdom and the United States showed an absolute reduction in false-positive results of 5.7 and 1.2% (US and UK, respectively) and in false-negative results of 9.4 and 2.7% compared to six independent radiologists. In the much more practical scenario of the AI supporting the work of medical professionals, it was able to reduce the workload of the 2nd reviewer by up to 88% when the images were evaluated using a “4-eyes principle” (2). Another study used AI in the detection and diagnosis of lung cancer in thoracic CT scans with an accuracy of 97% and a sensitivity of 94.4% in 420 cases obtained randomly from LIDC-IDRI database (4).

In pathology, Eckhardt et al. developed an AI program that could distinguish between AML samples and healthy controls based on bone marrow images with a very high sensitivity and specificity. Moreover, with the help of this AI application it was possible to predict with high probability the presence of one of the most common mutations in AML based on cytomorphological features alone (5).

Another large area of application for AI-assisted diagnostics is dermatology. Especially melanoma screening has seen several applications (6). The results from a study published in 2020 showed that an AI performed equally well as dermatologists in differentiating dermatoscopic close-ups of keratinizing malignancies of the skin and seborrheic keratoses as well as melanocytic nevi and melanomas (7, 8). Another study by Tschandl et al. showed that AI-assisted machine classification methods significantly outperformed the diagnostic accuracy of even clinical experts in the diagnosis of pigmented skin lesions (9). Another study, however, could show that dermatologists were still superior to the AI with regard to therapy decisions if anamnestic data (age, sex, and localization) were included (8).

Although most of these applications were usually retrospectively performed in intensively revised and annotated datasets, these cases show an increasing benefit and potential practical use of AI in the near future. Furthermore, they highlight the probable use of AI in medicine: as a tool to help clinicians make faster and better decisions, not to replace them, at least in the foreseeable future.

The Applications of AI in surgery can be divided into three categories: First, the preoperative phase, second, the intraoperative phase, and third, the postoperative phase (10). In addition to improved diagnostics and prognosis prediction, surgical risk stratification also play an important role in the preoperative phase (10). These processes should help rate an adapted risk profile before a surgical intervention and enable individual guidance in the choice of treatment. Bihorac et al. developed an AI application called “MySurgeryRisk” (11), that can predict 8 postoperative complications and the risk of death at 1, 3, 6, 12, and 24 months with relatively high accuracy (11). A follow-up study comparing showed that the MySurgeryRisk algorithm was superior in predicting postoperative complications compared to risk assessment by physicians, however the estimates of the surgeons significantly improved after intensive use of the program (12).

In the intraoperative phase, AI applications might help to optimize surgical skills, enable improved identification of intraoperative anatomy or control simple surgical instruments by recognizing surgical gestures (10). Fully autonomous surgery by robots might be possible in the far future. A study by Yang et al. could show a better improvement in basic surgical skills when participants were trained by specialists in conjunction with AI assisted practice sessions (13). In addition, AI systems can already be used to improve the visualization of intraoperative anatomy with the aid of augmented or virtual reality (14–16). Although despite several proof-of-concept studies with promising results, most research endeavors do not progress to translation into clinical routine, amongst other things due to the lack of registration accuracy in the real environment (14).

The use of autonomous surgical robots, which independently perform surgical procedures on humans by means of AI-controlled systems, remains a vision at the present time (17, 18). Some surgical fields, such as orthopedic surgery, have shown promising advances in the use of robotic devices for specific tasks: Active robotic devices can help perform more precise bone cuts for total knee arhtroplasty using preoperative CT imagery with very good results (19–21).

Other surgical disciplines, such as visceral surgery, are struggling with the challenges of soft tissue surgery: preoperative planning, preoperative imagery transfer onto the complicated intraoperative imaging including tissue tracking of deforming tissues and finally the precise execution of planned tasks with adaptable control strategies in these soft tissues. Furthermore, laparoscopic abdominal surgery requires high maneuverability, usually not found in current robotic surgery models. Nevertheless, the implementation of AI systems is progressing with above mentioned advances in intraoperative imagery and recognition (16), but also with first fully autonomus surgical robots that can perform specific tasks such as a laparoscopic intestinal anastomosis on a porcine model (22). In this specialized application, the AI robots outperformed expert surgeons manual technique in regard to needle placement corrections, suture spacing, suture bite size, completion time, lumen patency, and leak pressure.

Although the number of medical AI applications is steadily increasing, many ethical and medico-legal aspects have so far been treated only negligently and many ethical and medico-legal problems remain unresolved such as accountability, liability, and culpability (23). This is highly relevant, as it is precisely these points that will have a major influence on how high the acceptance and practical use of AI applications will be among doctors in the future. Despite these technical and ethical challenges, however, there is no doubt that the development and implementation of AI is advancing ever faster and will have a significant impact on the working lives of surgeons in the coming years.

Yet, little data is available on how surgeons understand and perceive AI applications in Germany and for which surgical applications they deem AI particularly relevant. Therefore, we conducted this survey among surgeons in Germany to explore their acceptance and openness toward AI as well as try to identify potential problems in their use in the surgical field. This survey may help to draw conclusions as to what extent AI applications are applicable to surgery and what the prerequisites are for their implementation into surgical routine.

## Methods

### Selection criteria for the survey collective

We conducted an anonymous survey using the web-based tool “LimeSurvey” in accordance with the General Data Protection Regulation (GDPR). Potential participants of the study were contacted by e-mail and asked to complete the questionnaire. Eligible participants were surgeons who currently practice or have practiced in Germany in one of the following surgical specialties: general surgery, visceral and oncological surgery, thoracic surgery, vascular surgery, neurosurgery, orthopedics and trauma surgery, and plastic surgery. In order to be able to conduct the survey with as homogeneous a group of practicing surgeons as possible, physicians with other specialties such as gynecology, urology, dermatology, otorhinolaryngology (ENT), or ophthalmology were excluded from the survey. Medical students who had already gained practical experience in the field of surgery but were not yet licensed at the time of the survey were also not addressed.

We conducted the survey between April 15, 2021 and December 15th, 2021. For this purpose, we contacted surgeons at the following hospitals or surgical centers: Dresden University Hospital, Heidelberg University Hospital, Ulm University Hospital, University Hospital “Rechts der Isar” of the Technical University of Munich, Mannheim University Hospital, Cologne University Hospital, Frankfurt University Hospital, Düsseldorf University Hospital, Rheinland Klinikum Neuss Lukaskrankenhaus, Evangelisches Krankenhaus Düsseldorf, Klinikum Itzehoe, Klinik Preetz—Krankenhaus des Kreises Plön, Städtisches Krankenhaus Kiel, Helios Klinikum Schleswig, Asklepios ASB Klinik Radeberg, Städtisches Klinikum Dresden, DRK Krankenhaus Mölln-Ratzeburg, Klinikum Nuernberg (Paracelsus Medical University). In addition, the survey was sent by e-mail to outpatient surgeons in Dresden. An approval of the local internal review board (IRB Dresden, IRB00001473) was not seeked, as it was not necessary for this type of research according to local regulatory guidelines and laws.

### Conception of the questionnaire

We have designed the questionnaire based on previous scientific surveys by Scheetz et al. (24), van Hoeck et al. (25), and Oh et al. (26). The questionnaire was in German and was comprised of 22 questions. Depending on the question type, single or multiple answers were possible. In questions 1–5, the participants are asked to provide general information about work experience, the environment of the workplace and age (categorized in decades) (“general question section”). Questions 6–22 evaluated what role the participating surgeons consider AI to play in their work environment and where they see risks and strengths regarding the use of AI in hypothetical scenarios. Participants had the option of not answering all questions in the survey and incomplete survey responses were included in the combined analysis (Supplementary Table 1).

### Statistical analysis

Data were analyzed using SPSS version 21 software (IBM Corp., Armonk, NY, USA).

Competitive analysis was performed to compare baseline characteristics between the groups of participants using chi-square test or Fisher's exact test for categorical variables. The American Association for Public Opinion Research guidelines were followed as applicable.

## Results

### Demographics

A total of 161 surgeons participated in the survey and 147 participants completed all survey questions. Respondents predominantly practiced as visceral/general surgeons (*n* = 89/55.27%) or in the field of Traumatology/Orthopedic surgery (*n* = 27/16.77%). Seventeen (10.56%) participants practiced as vascular surgeons, 8 (4.97%) respondents were neurosurgeons, 7 (4.34%) respondents worked in the field of plastic surgery. Five (3.1%) respondents were cardio-thoracic surgeons and 8 (4.96%) respondents did not specify their surgical specialty.

The vast majority of participants were employed either at a university hospital or at an academic teaching hospital [*n* = 123 (76.4%) and *n* = 27 (16.77%), respectively]. The median age of the participants was between 30 and 40 years. The median work experience amounted to 11–15 years. Further characteristics of the cohort in terms of demographics are shown in Table 1.

**Table 1:** Demographics of the participants addressed in question 1–5.

**Specialty**	**Number of participants**
General/visceral surgery	89 (55.27%)
Traumatology/orthopedics'	27 (16.77%)
Vascular surgery	17 (10.56%)
Neurosurgery	8 (4.97%)
Plastic surgery	7 (4.34%)
Cardio-thoracic surgery	5 (3.1%)
Non-specified	8 (4.96%)
**Place of employment**	
University hospital	123 (76.40%)
Academic hospital	27 (16.77%)
Primary care hospital	3 (1.86%)
Ambulatory health care	3 (1.86%)
Other health care provider	0 (0.00%)
Non-specified	5 (3.11%)
**Years of work experience**	
<5 years	39 (24.22%)
≥5–10 years	32 (19.88%)
≥11–15 years	37 (22.98%)
>15 years	47 (29.19%)
Non-specified	6 (3.73%)
**Age**	
<30 years	23 (14.29%)
≥30–40 years	67 (41.61%)
≥40–50 years	41 (25.47%)
>50 years	23 (14.29%)
Non-specified	7 (4.35%)
**Highest academic degree**	
License to practice medicine	45 (27.95%)
Specialist	59 (36.65%)
Assistant/full professor	38 (23.60%)
Master of Science	1 (0.62%)
PhD	9 (5.59%)
Others	2 (1.24%)
Non-specified	7 (4.35%)

### Current state of knowledge and experience in the use of AI in surgery

**Figure 1:**
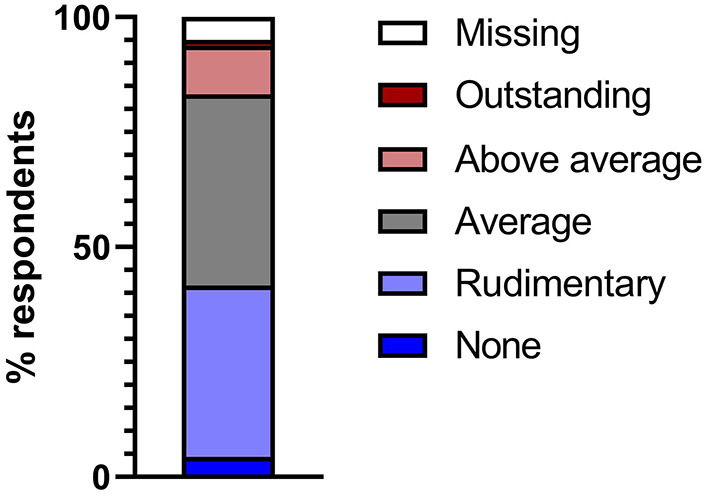
Self-reported knowledge of artificial intelligence and its application, relative to peers in that field.

A total of 85 (52.80%) participants stated that they were generally familiar with AI applications in medicine, and 69 (42.86%) participants responded that they had no experience with AI in the field of medicine so far. With regard to their own area of expertise, however, only 71 (44.1%) of the respondents indicated that they were aware of applications based on AI, while 83 (51.55%) of the participants denied any experience with AI in their own specialty. The vast majority of participants rated their knowledge of AI as average (*n* = 67; 41.61%) or below average (*n* = 60; 37.27%) relative to their peers. Only a minority claimed to have more experience than average with AI (*n* = 17; 10.56%) or considered themselves to be experts in the field of AI (*n* = 2; 1.24%) (Figure 1, Supplementary Table 1).

### Predicting the impact of AI on individual fields of expertise

**Figure 2:**
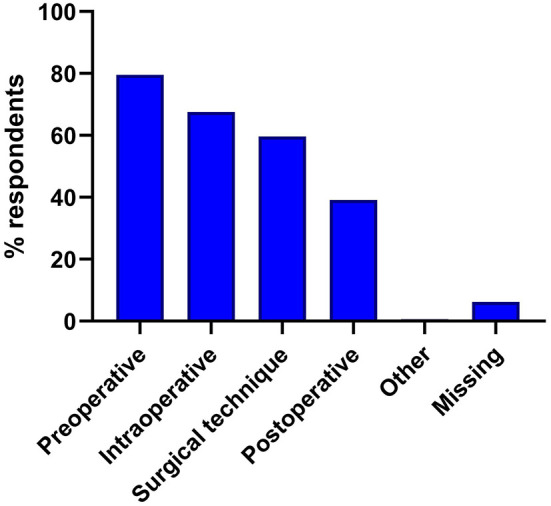
Perceived uses for artificial intelligence in medicine.

The vast majority (*n* = 139, 86.4%) of the respondents stated to be convinced that AI would play an important role in their field of specialty within the next 10 years. Almost half of the respondents (*n* = 76; 47.2%) claimed that this development will already have a significant influence in their specialty within the next 5–10 years and a further 21% (*n* = 34) assumed that this effect will already be evident at their workplace within the next 1–5 years (Supplementary Table 1). Overall, the participants of the survey stated to expect that AI would be most often employed for preoperative diagnostics/imaging, for intraoperative hybrid procedures and in the context of surgical techniques (Figure 2, Supplementary Table 1). At the same time, most participants did not expect the implementation of AI to have an impact on the number of staff required in their own area of work (Supplementary Table 1). Nevertheless, almost half of the respondents (*n* = 80; 49.69%) felt that their workplace was inadequately prepared for the introduction of AI into clinical practice. Accordingly, 69.57% (*n* = 112) also agreed with the statement that there should be more information and education available in their workplace/hospital about potential future applications of AI in their field of expertise (Supplementary Table 1).

### Potential applications of AI in clinical workflows in different fields of expertise

**Figure 3:**
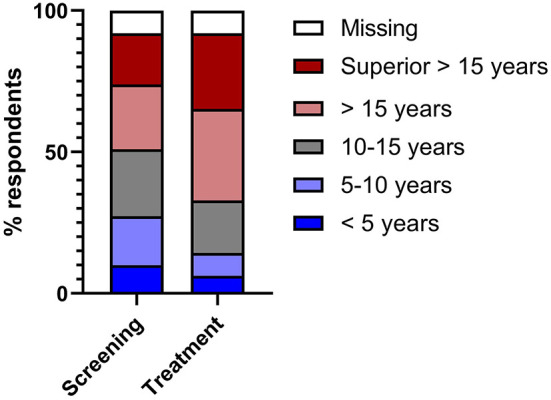
100% stacked bars showing the acceptable level of error for an AI tool used for disease screening (left) and clinical decision support (right).

According to the majority of respondents, the commercial use of AI is expected to first be implemented at university hospitals and specialized healthcare facilities. Only a small proportion of respondents held the opinion that AI would be used commercially in the public sector or in private clinics first (Supplementary Table 1). In the context of disease screening, most survey respondents considered that AI systems would need to achieve performance that was equal (*n* = 38, 23.60%) or even superior (*n* = 37, 22.98%) to the average performing specialist with 10–15 years of professional experience. A further 18.01% (*n* = 29) of respondents even believe that the failure rate for AI systems used for screening for diseases in their field of expertise should be superior to the average performance of a designated specialist (professional experience >15 years) (Figure 3, Supplementary Table 1). The expectations for the accuracy of AI used to support treatment decisions for diseases in the corresponding field (e.g., surgical indications, use of medications, complication management) were even more stringent (Figure 3, Supplementary Table 1). For this application, significantly more respondents claimed that the failure rate for AI should be equal or even superior to the average performance of a designated specialist with more than 15 years of experience [*n* = 52 (32.30%) and *n* = 43 (26.71%), respectively, *p* = 0.01, Table 2].

**Table 2:** Comparison of the acceptable level of error for an AI tool used for disease screening (question 17) and treatment decision support (question 18).

	**Question 17: In your opinion, what level of error is acceptable for artificial intelligence systems applied for the purpose of screening for diseases in your field (e.g., differential diagnosis of benign vs. malignant tumors, detection of fractures, detection of ischemic areas)?**	**Question 18: In your opinion, what level of error is acceptable for artificial intelligence systems used to make treatment decisions for diseases in your field (e.g., indication for surgery, use of medication, complication management)?**	***P*-value**
Professional experience <5 years	16	10	***P*** **=** **0.01**
Professional experience 5–10 years	28	13	
Professional experience 10–15 years	38	30	
Professional experience >15 years	37	52	
Superior to professional experience >15 years	29	43	

P-value evaluated by the Chi-square (χ^2^) test.

Subsequently, two hypothetical clinical workflows were proposed to the participants of the survey. In the first scenario, the participants were asked whether they would agree or disagree with the following workflow: “the clinical images of a patient are analyzed with AI. A designated specialist (professional experience >15 years) subsequently reviews both the image and the results of the AI and makes a diagnosis based on this.” According to our survey, 137 (85.09%) of the respondents would consider using this workflow whereas 8 (4.97%) respondents would refuse this AI application. For the second scenario, the respondents were asked if they would consider using the following clinical workflow: “A patient's pre-operative data is analyzed with AI. A designated specialist (professional experience >15 years) makes a treatment decision based on this information.” Compared to the first scenario, significantly fewer respondents [*n* = 112 (69.57%)] declared they would consider this AI application in a current clinical workflow (*p* < 0.001, Table 3).

**Table 3:** Comparison of the acceptable level of error for a clinical workflow using an AI tool for disease screening (question 19) and treatment decision support (question 19).

	**Question 19: Would you consider using the following clinical workflow: The clinical images of a patient are analyzed with artificial intelligence. A specialist (m/f/d) (professional experience >15 years) reviews both the image and the artificial intelligence results and makes a diagnosis based on them**.	**Question 20: Would you consider using the following clinical workflow? A patient's preoperative data is analyzed with artificial intelligence. A specialist (m/f/d) (professional experience >15 years) makes a therapy decision based on this information**.	
Yes	137	112	***P*** **<** **0.001**
No	8	27	
I cannot assess	2	8	

P-value evaluated by the Chi-square (χ^2^) test.

### Perceived advantages and concerns of the use of AI

**Figure 4:**
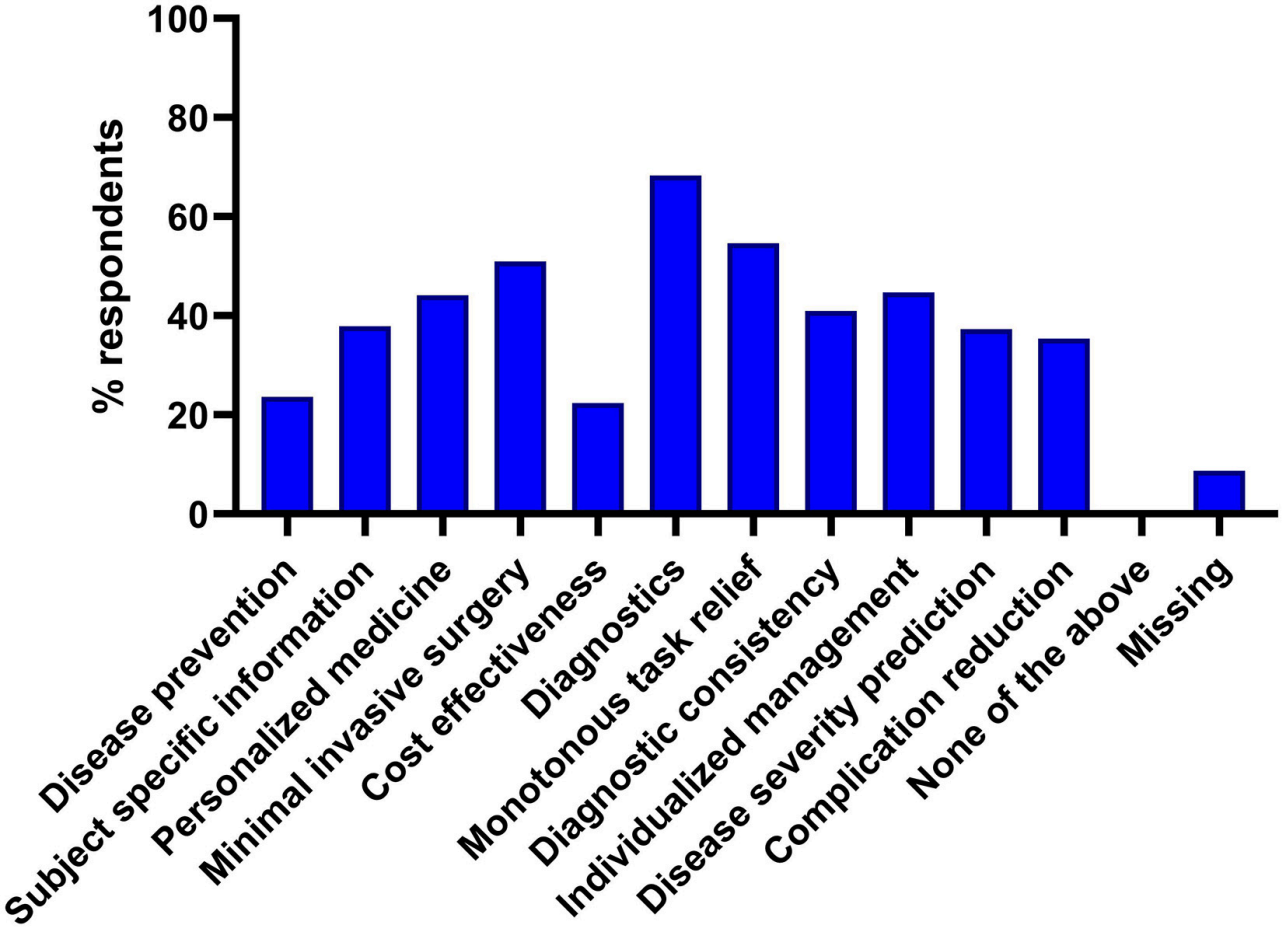
Perceived advantages of the use of artificial intelligence in medicine. Responses were selected from a list of set choices, multiple selections were possible.

**Figure 5:**
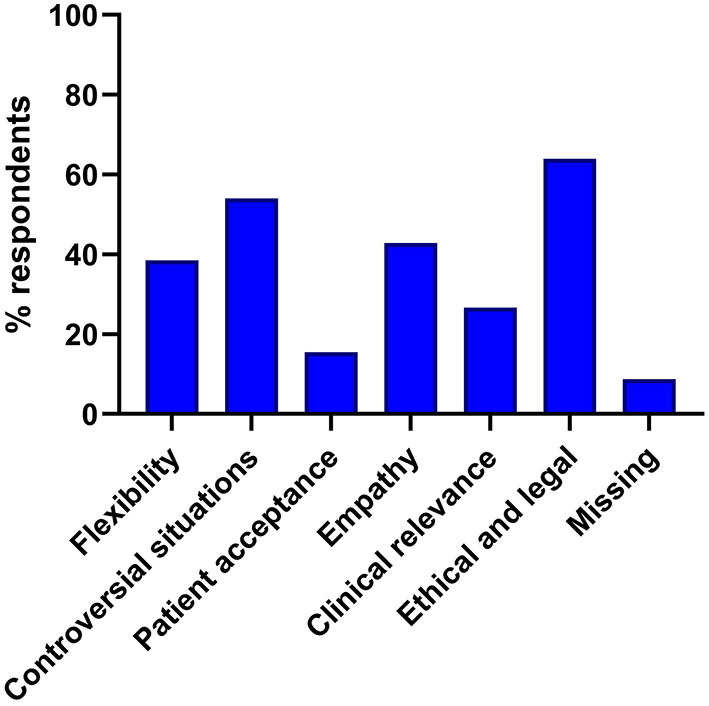
Perceived disadvantages of the use of artificial intelligence in medicine. Responses were selected from a list of set choices, multiple selections were possible.

Survey participants were asked to nominate which of a range of clinical applications of AI in their field of select application options, for which they would perceive the greatest potential advantages, and which are of most concern. The top three ranked potential advantages of AI were (1) improved diagnostic accuracy, (2) reduced time spent by specialists on monotonous tasks and (3) more precise and minimally invasive surgical techniques (Figure 4). The top three ranked potential concerns of AI were (1) ethical and legal problems regarding liability, (2) the applicability to controversial issues and (3) low ability to empathize and consider the patient's emotions (Figure 5).

## Limitations of the study

The survey in this publication is aimed exclusively at surgeons in Germany. Thus, the survey population is significantly more homogeneous than in previously published works. Although the survey was sent out to a variety of surgeons including university hospitals, academic teaching hospitals, primary care and outpatient hospitals, in the final evaluation, 82.8% of the participants stated that they were employed at a university hospital. However, the response rate of 37.5% in this survey was significantly higher than the survey rates of comparable studies by Scheetz et al. with 5.1–20.4% or Oh et al. with 22.3% (24, 26). Thus, the proportion of surgeons from this environment is clearly overrepresented, while the proportion of surgeons from non-university hospitals and outpatient facilities cannot be considered representative. Furthermore, the proportion of visceral surgeons was clearly overrepresented compared to traumatology and orthopedic surgeons. The median age of 30–40 years on the other hand does reflect the current age distribution in Germany quite well (27). We do expect the acceptance of AI applications to be higher in surgeons working in university hospitals, on the other hand, these surgeons will probably be the surgeons that will come into contact with practical AI implementations first. Nevertheless, the strength of the survey lies in the fact that, due to a relatively homogeneous cohort and a high response rate, it reflects a good picture of opinion as to the significance and acceptance of AI among selected surgeons in Germany.

## Discussion

AI is one of the key technologies of the twenty-first century and is expected to impact the professional and private lives of many people in the coming decades. In this context, not only advantages, but also risks or ethical areas of tension are increasingly coming into focus. Surgeons also face this discourse, because surgery is one of the medical domains that has benefited from rapid technological progress in recent decades. To date, the interest in and the acceptance of AI applications in the field of surgery have not been systematically investigated. We therefore conducted this survey to determine the approval of AI among surgeons in Germany in their working environment.

The majority of respondents indicated that they are familiar with applications for AI in medicine. Most participants self-classified their knowledge of AI in general. This self-assessment is consistent with the studies by Scheetz et al. and Oh et al. (24, 26). In these two surveys, most medical professionals stated that they had an “average” level of knowledge regarding AI. Of note, in our survey most of the responding surgeons claimed to be more familiar with AI applications in medicine in general than in their specialized surgical field. One possible explanation for this difference might be that currently the majority of AI applications are predominantly used for diagnostic procedures (2, 3, 5, 6, 28), which are primarily the domain of other medical specialties such as radiology, pathology or dermatology (2, 3, 5, 6, 28). Accordingly, it can be conjectured that many respondents of the survey perceive a great development and application potential in these disciplines in particular for the next decade.

However, even though many survey participants stated to believe that AI applications will become increasingly important within the next 10 years, they mentioned to experience substantial deficits in the implementation of AI applications in their own professional environment. Physicians from other specialties share these concerns: In the field of radiology, Yang et al. have reported that students and physicians believe that AI-literacy training during medical school and residency is inadequate (29). Accordingly, the respondents emphasized the need for education and training in AI (29). As a proposed solution, the authors suggest closer collaboration between IT specialists and physicians to improve the implementation of AI applications in the corresponding workplace (29).

Furthermore, we assessed the opinion of surgeons on the impact of AI applications on the development of staff and workplace requirements. This point is of particular relevance because the introduction of AI is often associated both with the creation of new jobs and with the disappearance of entire occupational segments. In this regard, many surgeons believed that their tasks could not be substituted by the application of AI.

In the context of working environment changes, Chockley and Emanuel have verbalized the provocative hypothesis that machine learning through AI was the ultimate threat that could spell the end of radiology as a thriving specialty in the next 5–10 years (30). Likewise, Pakdemirli has speculated that AI could reduce radiologists' jobs within the next few years (31). A survey by van Hoek et al. supports these opinions: here, radiologists were more likely than surgeons to expect loss of competence due to AI applications (25). This discrepancy may relate to the fact that surgery is a manual discipline that requires physical dexterity. Moreover, the data used by AI systems for image analysis, as is the basis of applications in radiology, is likely to be more standardized and readily available than that needed for surgical AI systems. Although some experts believe that machines might master surgical tasks superior to humans within three to four decades, these are so far only educated guesses (1). Implementation of core surgical skills such as operating by means of AI-controlled robots is, according to the current state of research, only possible in rudimentary form and only for selective applications (14). Although recent advances, such as the autonomous intestinal anastomosis in a porcine model shows that the possibility of autonomous surgery can be achieved (22), the extremely complex interplay of intraoperative imaging including tissue tracking of deforming tissues, the precise execution of planned tasks with adaptable control strategies and the required high maneuverability in visceral surgery will make an implementation very computationally intensive and challenging. Autonomous surgery by robots is therefore not likely in the near future.

In accordance with this opinion, most participants of our survey expected that the implementation of AI will have very little to some impact on the personnel requirements in their specialist area in the next 10 years, and that overall, the approximate staff requirement in the respective specialist areas will not change as a result of the increasing abundance of AI.

Moreover, the participants were asked about their assessment and acceptance of different application scenarios for AI. Here, we distinguished between the application scenario “diagnosis confirmation using AI” and “therapy decision using AI.” The survey revealed that the requirements varied depending on the application scenario. In the context of AI-advised diagnosis confirmation, most participants in the survey considered an error level that corresponds to the professional experience of a senior surgeon as sufficient. On the other hand, the respondents had significantly higher expectations regarding the quality of an AI-assisted therapy decision tool. Here, over half of the participants stated that the error level of a corresponding AI application should at least keep up with the error level of a designated specialist, or even be superior to it. These different expectations may be explained by the fact that “therapy decisions” are more directly associated with serious consequences for patients than those arising from “diagnosis confirmation.” Accordingly patient safety and medico-legal requirements are significantly higher if the immediate therapy is also influenced by an AI application. However, given that diagnostic decisions usually imply a therapeutic strategy and therefore influence the selection of treatment interventions, more specific scenarios should be investigated in the future. In addition, studies have shown the “human” are currently superior to AI applications for therapeutic decision-making if more information is available (8). Thus, expert knowledge also plays an essential role in therapeutic decisions, which is naturally also required as a benchmark when using an AI system.

This aspect is also echoed in the last part of the survey, in which participants were asked for which categories they would see the greatest potential advantage or greatest disadvantage in the use of AI systems. Congruent with the response pattern from the previous answers, the category “Improved diagnostic confidence” received the greatest level of support. This is in line with the above-mentioned assumption that AI applications are already being used to some extent in the clinic, particularly for diagnostic procedures (2, 3, 5, 6, 28) and are therefore much more widely accepted among surgeons than other AI tools. However, survey participants also see great potential in AI to facilitate “More precise and minimally invasive surgical techniques,” which was the second most selected use case. At the present time, however, this must be interpreted more as a future vision. The development of autonomously operating robots is very demanding and particularly difficult to learn through AI (10, 14), although first robots can perform autonomous tasks in an experimental setting (22).

The most frequently selected potential disadvantage of AI applications in surgery was “If complications occur, there are ethical and legal problems with regard to liability.” According to current expert opinions, this is one of the greatest challenges when it comes to establishing AI applications in medicine (23, 32). Before AI applications are used in surgery, it is therefore essential to legally define liability in the event of malpractice and responsibility for possible health consequences. This inherent problem of AI implementation in any health care sector is essential in surgery, since errors and mistakes have immediate and serious consequences for patients. The solution of this medico-legal problem will decide the fate of AI implementation in medicine.

One probable solution will be the cooperative effort of surgeons and assistive AI systems, supporting specialists in performing their tasks better, safer and more reproducibly. The setup of most studies, where AI is pitched against humans does not make sense in the medical field, at least not at the current time, much more realistic is an assisted approach, where AI systems support specialist by analyzing the increasing amount of data behind each single patient, improving preoperative diagnosis and therapy planning and supporting the intraoperative course by intraoperative imaging assistance as well as performance of specific tasks.

In conclusion, German surgeons are in principle positively disposed toward AI applications in their fields of practice. However, many surgeons see a deficit in the realization of AI applications in their own clinic. Accordingly, medical education programs targeting both medical students and healthcare professionals should convey basic knowledge about the development and clinical implementation process of AI applications in different medical fields including surgery. In addition, the public discourse needs to address the medico-legal and ethical framework conditions under which AI should be applied in the context of diagnostics and therapeutic decision making in surgery.

## Data availability statement

The raw data supporting the conclusions of this article will be made available by the authors, without undue reservation.

## Author contributions

MP, JS, and CK designed and drafted the survey, performed statistical analysis, and drafted the manuscript. CR, MD, FO, UB, and FK helped with the survey design, survey distribution, and data analysis. OS, PW, and JW supervised the survey, analyzed, and interpreted the data. CK and JS were responsible for the final draft and editing of the manuscript. All authors read and approved the final manuscript.

## Conflict of interest

The authors declare that the research was conducted in the absence of any commercial or financial relationships that could be construed as a potential conflict of interest.

## Publisher's note

All claims expressed in this article are solely those of the authors and do not necessarily represent those of their affiliated organizations, or those of the publisher, the editors and the reviewers. Any product that may be evaluated in this article, or claim that may be made by its manufacturer, is not guaranteed or endorsed by the publisher.
