# Supplementary material for: The use and future perspective of Artificial Intelligence—A survey among German surgeons
Source: Front Public Health. 2022 Oct 5;10:982335. doi: 10.3389/fpubh.2022.982335 (PMC9580562; doi:10.3389/fpubh.2022.982335)
Supplement: Supplementary file 1 [file Table_1.DOCX]

Supplementary Table 1

Results of the survey on questions 6 - 22. The questions were initially asked in German and translated into English for publication matters.

| **Question 6: Are you familiar with applications for artificial intelligence in medicine?** | | |
| --- | --- | --- |
| ***Answer*** | ***Number*** | ***Percent*** |
| Yes (Y) | 85 | 52.80% |
| No (N) | 69 | 42.86% |
| No answer | 0 | 0.00% |
| Not finished or not shown | 7 | 4.35% |
| **Question 7: Are you familiar with artificial intelligence applications in your field?** | | |
| ***Answer*** | ***Number*** | ***Percent*** |
| Yes (Y) | 71 | 44.10% |
| No (N) | 83 | 51.55% |
| No answer | 0 | 0.00% |
| Not finished or not shown | 7 | 4.35% |
| **Question 8: How would you rate your knowledge of artificial intelligence in general?** | | |
| ***Answer*** | ***Number*** | ***Percent*** |
| Outstanding expertise (1) | 2 | 1.24% |
| Above average (2) | 17 | 10.56% |
| Average (3) | 67 | 41.61% |
| Rudimentary (4) | 60 | 37.27% |
| no knowledge (5) | 7 | 4.35% |
| No answer | 0 | 0.00% |
| Not finished or not shown | 8 | 4.97% |
| **Question 9: Do you think that artificial intelligence will be used in your field in the next 10 years?** | | |
| ***Answer*** | ***Number*** | ***Percent*** |
| Yes (Y) | 139 | 86.34% |
| No (N) | 8 | 4.97% |
| No answer | 4 | 2.48% |
| Not finished or not shown | 10 | 6.21% |
| **Question 10: If you answered "yes" to question 9, in which area do you see the use of artificial intelligence (multiple answers possible)?** | | |
| ***Answer*** | ***Number*** | ***Percent*** |
| Preoperative diagnostics/imaging (1) | 128 | 79.50% |
| Intraoperative hybrid procedures (2) | 108 | 67.08% |
| Use in the surgical technique (3) | 96 | 59.63% |
| Postoperative therapy decisions (4) | 63 | 39.13% |
| Other | 1 | 0.62% |
| Not finished or not shown | 10 | 6.21% |
| **Question 11: How long do you think it will take for artificial intelligence to have a noticeable impact in your field?** | | |
| ***Answer*** | ***Number*** | ***Percent*** |
| Within 1 year (1) | 2 | 1.24% |
| 1-5 years (2) | 34 | 21.12% |
| 5-10 years (3) | 76 | 47.20% |
| > 10 years (4) | 37 | 22.98% |
| Never (5) | 1 | 0.62% |
| No answer | 0 | 0.00% |
| Not finished or not shown | 11 | 6.83% |
| **Question 12: Which area of healthcare do you think will be the first to use artificial intelligence commercially (multiple answers possible)?** | | |
| ***Answer*** | ***Number*** | ***Percent*** |
| Public primary care such as public health centres (1) | 16 | 9.94% |
| Primary care in private clinics (2) | 15 | 9.32% |
| Specialised clinics (tumour clinics. cardiology. spinal surgery) (3) | 92 | 57.14% |
| University Hospitals (4) | 116 | 72.05% |
| Other | 3 | 1.86% |
| Not finished or not shown | 12 | 7.45% |
| **Question 13: In your opinion, will the application of artificial intelligence have an impact on staffing needs in your field over the next ten years?** | | |
| ***Answer*** | ***Number*** | ***Percent*** |
| To a large extent (1) | 5 | 3.11% |
| Somewhat (2) | 44 | 27.33% |
| Very little (3) | 69 | 42.86% |
| Not at all (4) | 15 | 9.32% |
| I cannot assess (5) | 16 | 9.94% |
| No answer | 0 | 0.00% |
| Not finished or not shown | 12 | 7.45% |
|  |  |  |
| **Question 14: In which direction will the demand for workers in your field change due to artificial intelligence?** | | |
| ***Answer*** | ***Number*** | ***Percent*** |
| Increase (1) | 24 | 14.91% |
| Decrease (2) | 33 | 20.50% |
| No change (3) | 61 | 37.89% |
| I cannot assess (4) | 31 | 19.25% |
| No answer | 0 | 0.00% |
| Not finished or not shown | 12 | 7.45% |
| **Question 15: Do you think that your workplace/clinic is adequately equipped for the introduction of artificial intelligence in your field?** | | |
| ***Answer*** | ***Number*** | ***Percent*** |
| Yes (1) | 41 | 25.47% |
| No (2) | 80 | 49.69% |
| I cannot assess (3) | 28 | 17.39% |
| No answer | 0 | 0.00% |
| Not finished or not shown | 12 | 7.45% |
| **Question 16: Should there be a better information availability at your workplace/clinic (e.g. training, courses) about the possible future application of artificial intelligence in your field?** | | |
| ***Answer*** | ***Number*** | ***Percent*** |
| Yes (1) | 112 | 69.57% |
| No (2) | 18 | 11.18% |
| I cannot assess (3) | 19 | 11.80% |
| No answer | 0 | 0.00% |
| Not finished or not shown | 12 | 7.45% |
| **Question 17: In your opinion, what level of error is acceptable for artificial intelligence systems applied for the purpose of screening for diseases in your field (e.g. differential diagnosis of benign vs. malignant tumours, detection of fractures, detection of ischaemic areas)?** | | |
| ***Answer*** | ***Number*** | ***Percent*** |
| Equivalent to the average performance of an assistant physician (m/f/d) (professional experience < 5 years) (1) | 16 | 9.94% |
| Equivalent to the average performance of a medical specialist (m/f/d) (professional experience approx. 5 - 10 years) (2) | 28 | 17.39% |
| Equivalent to the average performance of a senior physician (m/f/d) (professional experience approx. 10 - 15 years) (3) | 38 | 23.60% |
| Equivalent to the average performance of a proven specialist (m/f/d) (professional experience > 15 years) (4) | 37 | 22.98% |
| Superior to the average performance of a proven specialist (m/f/d) (professional experience > 15 years) (5) | 29 | 18.01% |
| No answer | 0 | 0.00% |
| Not finished or not shown | 13 | 8.07% |
|  |  |  |
| **Question 18: In your opinion, what level of error is acceptable for artificial intelligence systems used to make treatment decisions for diseases in your field (e.g. indication for surgery, use of medication, complication management)?** | | |
| ***Answer*** | ***Number*** | ***Percent*** |
| Equivalent to the average performance of an assistant physician (m/f/d) (professional experience < 5 years) (1) | 10 | 6.21% |
| Equivalent to the average performance of a medical specialist (m/f/d) (professional experience approx. 5 - 10 years) (2) | 13 | 8.07% |
| Equivalent to the average performance of a senior physician (m/f/d) (professional experience approx. 10 - 15 years) (3) | 30 | 18.63% |
| Equivalent to the average performance of a proven specialist (m/f/d) (professional experience > 15 years) (4) | 52 | 32.30% |
| Superior to the average performance of a proven specialist (m/f/d) (professional experience > 15 years) (5) | 43 | 26.71% |
| No answer | 0 | 0.00% |
| Not finished or not shown | 13 | 8.07% |
|  |  |  |
| **Question 19: Would you consider using the following clinical workflow: The clinical images of a patient are analysed with artificial intelligence. A specialist (m/f/d) (professional experience > 15 years) reviews both the image and the artificial intelligence results and makes a diagnosis based on them.** | | |
| ***Answer*** | ***Number*** | ***Percent*** |
| Yes (1) | 137 | 85.09% |
| No (2) | 8 | 4.97% |
| I cannot assess (3) | 2 | 1.24% |
| No answer | 0 | 0.00% |
| Not finished or not shown | 14 | 8.70% |
| **Question 20: Would you consider using the following clinical workflow? A patient's preoperative data is analysed with artificial intelligence. A specialist (m/f/d) (professional experience > 15 years) makes a therapy decision based on this information.** | | |
| ***Answer*** | ***Number*** | ***Percent*** |
| Yes (1) | 112 | 69.57% |
| No (2) | 27 | 16.77% |
| I cannot assess (3) | 8 | 4.97% |
| No answer | 0 | 0.00% |
| Not finished or not shown | 14 | 8.70% |
| **Question 21: For which of the following points do you see the greatest potential advantage in using artificial intelligence systems in your field? (Multiple answers possible)** | | |
| ***Answer*** | ***Number*** | ***Percent*** |
| Improved patient access to disease prevention (1) | 38 | 23.60% |
| Improved information access to subject-specific literature (2) | 61 | 37.89% |
| Personalised medicine (3) | 71 | 44.10% |
| More precise and minimally invasive surgical techniques (4) | 82 | 50.93% |
| More cost-effective health care (5) | 36 | 22.36% |
| Improved diagnostic certainty (6) | 110 | 68.32% |
| Less time spent by specialists on monotonous tasks (7) | 88 | 54.66% |
| Greater consistency in diagnostic and management decisions (8) | 66 | 40.99% |
| More individualised and evidence-based disease management (9) | 72 | 44.72% |
| Improved prediction of disease progression (10) | 60 | 37.27% |
| Reduction of postoperative complications (11) | 57 | 35.40% |
| None of the above (12) | 1 | 0.62% |
| Not finished or not shown | 14 | 8.70% |
| **Question 22: For which of the following do you see the greatest potential disadvantage in using artificial intelligence systems in your field? (Multiple answers possible)** | | |
| ***Answer*** | ***Number*** | ***Percent*** |
| It is not flexible enough to be applied to every patient (1) | 62 | 38.51% |
| It is difficult to apply it to controversial issues (2) | 87 | 54.04% |
| No acceptance by the patients (3) | 25 | 15.53% |
| Poor ability to empathise and consider the patient's emotional well-being (4) | 69 | 42.86% |
| It is developed by a specialist with little clinical experience in medical practice (5) | 43 | 26.71% |
| If complications occur, there are ethical and legal problems regarding liability (6). | 103 | 63.98% |
| Not finished or not shown | 14 | 8.70% |
